# Characterizing zero-dose and under-vaccinated children among refugees and internally displaced persons in the Democratic Republic of Congo

**DOI:** 10.1186/s40794-024-00225-0

**Published:** 2024-07-15

**Authors:** Marcellin Mengouo Nimpa, Aimé Cikomola Mwana-Wabene, John Otomba, Jean-Crispin Mukendi, M. Carolina Danovaro-Holliday, Franck-Fortune Mboussou, Dieudonné Mwamba, Leandre Kambala, Dolla Ngwanga, Cedric Mwanga, Sume Gerald Etapelong, Issaka Compaoré, Moise Désiré Yapi, Daniel Katuashi Ishoso

**Affiliations:** 1 World Health Organization (WHO) Country Office, Kinshasa, Democratic Republic of Congo; 2 Expanded Program of Immunization, Kinshasa, Democratic Republic of Congo; 3 Immunization, Analytics and Insights (IAI), Department of Immunization, Vaccines and Biologicals (IVB), World Health Organization (WHO), Geneva, Switzerland; 4 World Health Organization African Regional Office, Brazzaville, Republic of the Congo; 5 National Institute of Public Health, Kinshasa, Democratic Republic of the Congo; 6 Kinshasa School of Public Health, University of Kinshasa, Kinshasa, Democratic Republic of Congo; 7 Immunization, Vaccine-Preventable Diseases and Polio Transition (IVP) Unit, Department of Communicable Diseases (DCD), WHO Regional Office for the Eastern Mediterranean (EMRO), Cairo, Egypt; 8 Associés en Management public et Développement (AMD) International, Ouagadougou, Burkina Faso

**Keywords:** Democratic Republic of Congo (DRC), Refugees and internally displaced persons (IDPs), Zero-dose, Under-vaccinated, Reasons, Behavioral and social drivers

## Abstract

**Background:**

The Democratic Republic of Congo (DRC) has one of the highest numbers of un and under-vaccinated children as well as number of refugees and internally displaced persons (IDPs) in the world. This study aims to determine and compare the proportion and characteristics of zero-dose (ZD) and under-vaccinated (UV) children among refugees and IDPs in the DRC, as well as the reasons for incomplete vaccination schedules.

**Methods:**

Data from a rolling vaccination coverage survey conducted from September 10, 2022, to July 03, 2023, among refugees and IDPs in 12 provinces of the DRC. ZD was defined as a child aged 12–23 months who had not received any dose of pentavalent vaccine DTP-Hib-Hep B (by card or recall) and UV as a child who had not received the third dose of pentavalent vaccine. The proportions of non and under-vaccination and the associated factors using a logistic regression model are presented for ZD and UV children. The reasons for non-vaccination of these children are described using the WHO-Immunization behavioral and social-drivers-conceptual framework and compared using Pearson’s Chi2 test.

**Results:**

Of 692 children aged 12 to 23 months included in the analysis, 9.3% (95% CI: 7.2–11.7%) were ZD and 40.9% (95% CI: 95%: 37.2–44.6%) UV. The Penta1/Penta3 drop-out rate was 34.9%. After adjustment, ZD children had a significant history of home or road birth. And UV children were significantly associated with mothers/caregivers being under 40, uneducated, farmers, ranchers, employed, rural residents, as well as with home or road births. Reasons linked to people’s perceptions and feelings were cited much more often for ZD (50.0%) than for UV (38.3%). Those related to social reasons were cited much more often by ZD (40.6%) than by UV (35.7%). Reasons related to “programmatic and practical issues” were cited less for ZD (90.5%) than for UV (97.1%).

**Conclusions:**

ZD and UV children represent significant proportions in refugee and IDPs sites in the DRC. However, the proportion of ZD is less than for the entire country, while the proportion of UV is comparable, reflected in a very high drop-out rate. Similarly to studies in the general population in DRC, the reasons for ZD children were mainly linked to challenges in caregiver motivation to vaccinate, while for UV children, they were more often linked to pro-grammatic and practical problems of the health system.

## Abstract (plain language summary)

The Democratic Republic of Congo (DRC) has one of the highest numbers of un and under-vaccinated children as well as number of refugees and internally displaced persons (IDPs) in the world. This study aims to determine and compare the proportion and characteristics of zero-dose (ZD) and under-vaccinated (UV) children among refugees and IDPs in the DRC, as well as the reasons for incomplete vaccination schedules. Data from a rolling vaccination coverage survey conducted from September 10, 2022, to July 03, 2023, among refugees and IDPs in 12 provinces of the DRC. ZD was defined as a child aged 12–23 months who had not received any dose of pentavalent vaccine DTP-Hib-Hep B (by card or recall) and UV as a child who had not received the third dose of pentavalent vaccine. The proportions of non and under-vaccination, the associated factors and reasons for non-vaccination are presented for ZD and UV children. Of 692 children aged 12 to 23 months included in the analysis, 9.3% (95% CI: 7.2–11.7%) were ZD and 40.9% (95% CI: 95%: 37.2–44.6%) UV. The Penta1/Penta3 drop-out rate was 34.9%. After adjustment, ZD children had a significant history of home or road birth. And UV children were significantly associated with mothers/caregivers being under 40, uneducated, farmers, ranchers, employed, rural residents, as well as with home or road births. Reasons linked to people’s perceptions and feelings were cited much more often for ZD (50.0%) than for UV (38.3%). Those related to social reasons were cited much more often by ZD (40.6%) than by UV (35.7%). Reasons related to “programmatic and practical issues” were cited less for ZD (90.5%) than for UV (97.1%). ZD and UV children represent significant proportions in refugee and IDPs sites in the DRC. However, the proportion of ZD is less than for the entire country, while the proportion of UV is comparable, reflected in a very high drop-out rate. Similarly to studies in the general population in DRC, the reasons for ZD children were mainly linked to challenges in caregiver motivation to vaccinate, while for UV children, they were more often linked to pro-grammatic and practical problems of the health system.

## Background

Routine immunization is one of the best and most cost-effective investments to save lives, promote good health and well-being and contributes to the achievement of the Sustainable Development Goals (SDGs) to “leave no one behind” [1].

The effect of vaccination to reduce and control epidemics is observed when the density of the target populations covered by a vaccine is high, known as the Herd Immunity Threshold concept [2]. This coverage would be influenced by humanitarian crises, health crises or natural disasters which can lead to outbreaks of vaccine preventable diseases [3]. Zero-dose children (ZD) are children who did not receive any dose of vaccine containing diphtheria, pertussis, and tetanus (DTP) [4]. They are found in many communities, especially in urban slums, conflict affected areas, and remote, hard-to-access rural areas [1, 4]. The number of ZD in-creased from 12.9 million in 2019 to 18.1 million in 2021 worldwide then decreased to 14.3 million in 2022 [5]. The number of under vaccinated children (UV), defined as children who have not received the third dose of diphtheria, pertussis, and tetanus (DPT3) vaccine [4], increased from 19 million in 2019 to 22.7 million in 2020 and then de-creased to 21.4 million in 2022 worldwide [5, 6]. Gavi, the Vaccine Alliance, through its strategic plan, aims to reduce by 25% the number of children who have not received any vaccine by 2025 and, in line with the 2030 Immunization Agenda (IA2030), to reduce the number of ZDs by 50% or more by 2030 [7].

The Democratic Republic of Congo (DRC) is one of the countries where routine vaccination coverage remains below the 90% target recommended in the 2030 Immunization Agenda. Indeed, the number of ZD and UV children in this country re-mains one of the highest in Africa and the world [1, 8, 9]. According to the household vaccination coverage survey conducted by the Kinshasa Public Health School (KSPH) in 2021, approximately 19.1% of children between 12 and 23 months had never been vaccinated with DTP-containing vaccine, representing about 771,098 ZD children in one birth cohort. One in four (25.5%) of the children aged 12–23 months did not receive DTP3, representing approximately 1,029,476 UV children [10]. These children are therefore at high risk of contracting related vaccine-preventable diseases (VPDs) [9]. In 2023, there were 522,000 refugees and 6.9 million internally displaced persons (IDP) in DRC due to multiple humanitarian crisis [11].

National authorities are committed to paying particular attention to these special populations not only on the traditional provision of vaccination services, but also on vaccination against COVID-19. Routine vaccination campaigns and supplementary vaccination activities (SIAs) were carried out in 12 provinces hosting refugees and internally displaced persons between July and August 2022.Following these campaigns, a vaccination coverage survey (VCS) was conducted in the 12 provinces hosting refugees and IDPs out of the 26 in the country to assess the level of routine vaccination coverage of children aged 6 to 23 months, women who have given birth in the last twelve months, and the functionality of vaccination services at facilities and villages. This secondary analysis carried out on data from this VCS aims to determine and compare certain characteristics of ZD and UV children among refugees and IDPs in the DRC. We also looked as the reasons for the non-completeness of the vaccination schedule in order to guide the strategic implementation of the EPI and improve vaccine equity among vulnerable communities.

## Methods

### Setting

The Democratic Republic of Congo (DRC) is a large country located in the central part of Africa, with a population of approximately 115.7 million in 2021. There are 4,037,161 surviving infants [10]. The health system has three levels (national, intermediate, and peripheral) and routine vaccination is an essential preventive intervention implemented at health facilities [8].

### Study design

This study is an analytical cross-sectional study in which we reviewed DRC vaccination coverage survey data collected from September 10, 2022, to July 3, 2023. Data collection took place in twelve of thirteen provinces planned, namely Bas Uele, Haut Uele, Ituri, Kasaï-Central, Kasaï-Oriental, Lomami, Maniema, North Kivu, South Kivu, Tanganyika and Tshopo. Kongo-Central province was excluded because the data collector declared target was not found at the indicated location.

### Sampling, target populations and data sources

We used the World Health Organization (WHO) approach to routine immunization coverage surveys [12]. The sampling for this study was stratified for all provinces that had IDPs and refugees based on the information available. At the level of Health Zones (Health Districts), the sampling included clusters with weighted random selection (proportional to size) of Health Districts for each province using the random function of the Excel software and taking the first value that appeared after filtering by decreasing order of magnitude. With level 1 (national: A) and complete vaccination coverage in children aged 12–23 months of 41.5%, the K was equivalent to 1, the degree of precision was set at 10% and the confidence level at 95%. As information on intra-cluster correlation was not available, the highest value of 1/3 with the weighted covariance coefficient (CVw) of 0.5 was considered for an unmatched variance term of the effect of plan of 1.22 (1 + CVw2).

The effective sample size of 102 was calculated by this quantity (B):

$$\varvec{n} \ge \frac{\varvec{K}\times {\varvec{Z}}_{1-\frac{\propto }{2}}^{2}}{4\times {\varvec{d}}^{2}} + \frac{1}{\varvec{d}} -2\times {\varvec{Z}}_{1-\frac{\propto }{2}}^{2} + \frac{{\varvec{Z}}_{1-\frac{\varvec{\alpha }}{2} }+2}{\varvec{K}}$$

The design effect of 2.9 was calculated by this quantity (C):

$$\varvec{E}\varvec{P}\varvec{S}=\left[1+\left(\varvec{m}-1\right)\times \varvec{C}\varvec{C}\varvec{I}\right]\times [1+ {\varvec{C}\varvec{V}\varvec{w}}^{2}]$$

It was accepted that each 5 households would produce an eligible child, i.e., 20% of the households visited (D) and the rates of refusal or unavailability of respondent set at 10% for an increase of 11% in the effective sample size. (E). The expected average respondent per cluster was set at 7 (m).

Total respondents who completed the questionnaires was estimated as A*B*C or 298.

The number of households to visit to reach the size of respondents is estimated by A*B*C*D*E, i.e., 1,641 households.

The number of clusters (Health Districts) is obtained by A*B*C/m or 43 clusters.

After weighting the Health Districts with refugees or internally displaced persons (IDPs), 28 clusters were selected for areas with IDPs and 15 clusters for areas with refugees. Households were selected in a simple random manner on Excel with the random function () after their enumeration and indexing whether they are in communities, in refugee camps or IDP sites. All children aged 6–23 months with at least one parent or guardian who is an IDP or refugee who spent at least one night in the selected households were selected. The analysis presented here is restricted to children aged 12–23 months (see below).

### Definition of key variables

The following operational definition were used:

ZD: Zero-dose children were defined those children aged between 12 and 23 months at the time of survey who had not received any dose of pentavalent (vaccine against diphtheria, pertussis, tetanus, B hepatitis and Hemophilus influenzae type b) verified using card or other document, or according to caregiver recall.

UV: Under-vaccinated children were defined as those who had not received the third dose of pentavalent vaccine, while the first dose had been received, also based on documented evidence or caregiver recall.

Dropout rate: corresponds to the proportion of vaccinated children who have not completed their vaccination schedule. In this study, it refers to the difference in rates between the first and third doses of pentavalent vaccine.

### Data collection and tools

Data was collected by trained study staff (doctors, nurses, sociologists, etc.) using a standard WHO immunization VCS questionnaire containing socio demographic variables and ZD and UV as main outcome variables. The WHO behavioral and social drivers of vaccination (BeSD) framework to assess non-vaccination was also used [13].

The independent variables for this study were the area of residence (urban and rural), the location of the respondent (refugee camp, IDP/refugee sites and family or host house), belonging to special populations (IDP or refugees), caregiver or guardian’s sex, age, marital status (in a relationship or not in a relationship) and education (uneducated, primary and secondary or higher), head household occupation (unemployed, farmer or breeder and public or private employee) religion, understanding of local languages, sex and age of the child in months, and reasons for non-vaccination of the children concerned.

Regarding the reasons for non-vaccination, the BeSD framework recommended by WHO and global partners for analysis related to immunization was used as a reference [13]. This conceptual framework separates determinants of vaccination into four categories: category 1 relates to people’s thinking and feelings, category 2 relates to the social processes “family norms”, category 3 focuses on the motivation factors or willingness to be vaccinated and category 4 relates to programmatic and practical factors. Category 4 is further stratified into three groups: (1) geographical barriers, (2) interpersonal relational factors of health workers and (3) organizational barriers for immunization services.

Variable related to category 1 (thinking and feeling) included the fear of side effects, that related to category 2 (social processes “family norms”) included family problems, relegating the importance of child vaccination. Category 3 (motivation) did not have data. In category 4 (practical factors), the variable relating to geographical barriers included vaccination site being too far away. Interpersonal relational factors of health workers included poor reception of families by health workers. Those relating to organization of immunization services barriers included vaccination site not known by families, long wait, vaccination schedule not respected and vaccines frequently out of stock.

Information was collected by trained teams composed of composed of doctors and nurses, using a questionnaire configured in Kobo Collect.

### Data analysis

All statistical analysis were performed using STATA software, version 17, taking into account the complex sampling approach, and considering the 5% as the significance threshold.

Descriptive statistics was used to describe the proportion and characteristics of ZD and UV children among refugees and internally displaced persons. The Pearson’s Chi-square test was performed to compare the proportions of categorical variables when the minimum expected was ≥ 5%.

First, a bivariate analysis was performed, then a logistic regression model was established for refugees and internally displaced persons. Measures of association between each independent variable and a refugee child’s UV status were reported as odds ratios accompanied by their 95% confidence intervals. The logistic regression model was established using an automatic selection of “FORWARD” type variables and an entry probability of 0.05. The final model included only those variables whose effects remained significant after adjustment. The logistic regression was only carried out after verification of fit using the Hosmer-Lemeshow test.

In accordance with the “behavioral and social driver framework” model, the characteristics underlying the motivation of the mother/guardian to have the child vaccinated were first described as a percentage. These characteristics were then divided into two groups, namely: what people think and feel and social processes. Finally, the characteristics related to “practical questions” were described in percentage and according to the categories that relate to them.

### Ethical considerations

The protocol for this study was approved by the National Health Ethics Committee (CNES) of the Ministry of Public Health, Hygiene and Prevention under number n°420/CNES/BN/PMMF/2023 of January 10, 2023. Authorization was also granted by the health and political-administrative authorities. Before starting the interview, oral informed consent was obtained from the study participants. The research team provided the respondent with information on the nature of the study, its objectives, the risks, and benefits involved, the freedom to participate or not without any prejudice, confidentiality and contact details of the person in charge of the study for further contact if necessary. The study did not involve sample collection of any type. Confidentiality was respected by anonymizing the dataset.

## Results

### Description of the Sample

Table 1 describes the characteristics of the sample of 692 children aged 12–23 months. Caregivers/guardians were mostly aged 20 to 39, married or in stable partnership (76.0%), had not attended school (38.4%) or had completed primary school (42.5%), were mainly farmer/breeder (65.5%), Christians (91.5%) and lived in rural geo-graphical entities (94.4%). Slightly over half of children aged 12 to 23 months were boys (51.2%) and a majority had been born in a health facility (90.2%).

**Table 1:** Socio demographic characteristics of study population

Variables		*n* (%)
Place of residence		692 (100.0)
	In community	170 (24.6)
	In a refugee camp	162 (23.4)
	In the IDP home site	360 (52.0)
Mother/caregiver’s Age Group		692 (100.0)
	Under 20	54 (7.8)
	20–29 years old	335 (48.4)
	30–39 years old	204 (29.5)
	40–49 years old	74 (10.7)
	50 years and over	25 (3.6)
Mother/caregiver’s marital status		692 (100.0)
	Single	65 (9.4)
	Divorced/widowed	115 (16.6)
	Married	512 (74.0)
Mother/caregiver’s educational level		692 (100.0)
	Uneducated	266 (38.4)
	Primary	294 (42.5)
	Secondary/tertiary	132 (19.1)
Mother/caregiver’s profession		692 (100.0)
	Unemployed	122 (17.6)
	Farmer/Breeder	453 (65.5)
	Employee	117 (16.9)
Mother/caregiver’s religion		692 (100.0)
	None/traditional	24 (3.5)
	Christian	633 (91.5)
	Muslim	35 (5.1)
Place of residence		692 (100.0)
	Urban	39 (5.6)
	Rural	653 (94.4)
Sex of child		692 (100.0)
	Male	354 (51.2)
	Female	338 (48.8)
Place of birth of the child		692 (100.0)
	At home/on the road	68 (9.8)
	Health facility	624 (90.2)

With n = number of subjects in the sample

### Proportion of ZD and under vaccinations among refugee and internally displaced children

The percentage of ZD children aged 12 to 23 months was 9.3% (95% CI: 7.2 to 11.7%), and that of UV was 40.9% (95% CI: 95%: 37.2 to 44.6%) (Table 2). The penta1/penta3 drop-out rate was of 34.9% (Table 2).

**Table 2:** Proportion of Zero-Dose and Under-Vaccinated Children

	*n*	% (95%CI)
Overall sample	692	
ZD Children	64	9.3 (7.2–11.7)
UV Children	283	40.9 (37.2–44.6)

With n = number of subjects in the sample; ZD = Zero-Dose; UV = Under-Vaccinated

### Factors associated with ZD and UV among refugee and internally displaced children

After adjusting for independent variables, being ZD was only significantly associated with the place of birth of the child. However, children born at home or on the road had 7 times higher odds of being ZD than those born in a health facility (AOR = 7.45 (95% CI 3.88 to 14.32)).

For UV children, after adjusting for independent variables, this status was significantly associated with caregiver/guardians being below 40 years (30–39 years old AOR = 3.48 (95% CI 1.35 to 9.01), 20–29 years old AOR = 3.18 (95% CI 1.25 to 8.07)); the lack of maternal education (AOR = 1.90 (95% CI 1.33 to 2.71); the professional occupation of the caregiver (farmer/breeder AOR = 2.13 (95% CI 1.37 to 3.31) and employee AOR = 2.34 (95% CI 1.34 to 4.07)) compared to the unemployed; rural residence (AOR = 2.30 (95% CI 1.09 to 4.84)); as well as births at home or on the road (AOR = 2.35 (95% CI 1.35 to 4.09)) compared to births in health facilities (Tables 3 and 4).

**Table 3:** Bivariate and multivariate analysis of the association “zero-dose” and socio-demographic and economic characteristics, Vaccination Coverage Survey among refugees and IDPs, DRC

Variables		Zero-doses		Bivariate					Multivariate				
		Yes	No	OR	IC95%			*p*-value	AOR	IC95%			*p*-value
Place of residence								0.2096					
	*In community in a household*	20	150	1									
	*In a refugee camp*	10	152	0.49	*0.22*	*to*	*1.09*						
	*In the PDI home site*	34	326	0.78	*0.44*	*to*	*1.40*						
Mother/caregiver’s Age Group								0.8066					
	*Under 20*	4	50	1									
	*20–29 years old*	35	300	1.46	*0.50*	*to*	*4.28*						
	*30–39 years old*	16	188	1.06	*0.34*	*to*	*3.32*						
	*40–49 years old*	6	68	1.10	*0.30*	*to*	*4.12*						
	*50 years and over*	3	22	1.71	*0.35*	*to*	*8.27*						
Mother/caregiver’s marital status								0.418					
	*Single*	8	57	1									
	*Divorced/widowed*	13	102	0.91	*0.36*	*to*	*2.32*						
	*Married*	43	469	0.65	*0.29*	*to*	*1.46*						
Mother/caregiver’s educational level								0.4717					
	*Uneducated*	26	240	1									
	*Primary*	23	271	0.78	*0.44*	*to*	*1.41*						
	*Secondary/tertiary*	15	117	1.18	*0.60*	*to*	*2.32*						
Mother/caregiver’s profession								0.9943					
	*Unemployed*	11	111	1									
	*Farmer/Breeder*	42	411	1.03	*0.51*	*to*	*2.07*						
	*Employee*	11	106	1.05	*0.44*	*to*	*2.52*						
Mother/caregiver’s religion								**0.0064**					
	*None/traditional*	4	20	1									
	*Christian*	52	581	0.45	*0.15*	*to*	*1.36*						
	*Muslim*	8	27	1.48	*0.39*	*to*	*5.61*						
Place of residence								0.428					
	*Urban*	5	34	1									
	*Rural*	59	594	0.68	*0.25*	*to*	*1.79*						
Sex of child								0.1319					
	*Male*	27	327	1									
	*Female*	37	301	1.49	*0.89*	*to*	*2.51*						
Place of birth of the child								**< 0.001**					
	*Health facility*	23	45	1					1				
	*At home/on the road*	41	583	7.27	*4.01*	*to*	*13.16*		7.45	*3.88*	*to*	*14.32*	**< 0.001**

**Table 4:** Bivariate and multivariate analysis of the association “under-vaccinated” and socio-demographic and economic char-acteristics, Vaccination Coverage Survey among refugees and IDPs, DRC

		Under-vaccinated		Bivariate					Multivariate				
		Yes	No	OR	IC95%			*p*-value	AOR	IC95%			*p*-value
Place of residence								**0.0003**					
	*In community in a household*	96	74	1									
	*In a refugee camp*	59	103	0.44	*0.28*	*to*	*0.69*						
	*In the PDI home site*	192	168	0.88	*0.61*	*to*	*1.27*						
Mother/caregiver’s Age Group								**0.0365**					
	*50 years and over*	7	18	1					1				
	*40–49 years old*	29	45	2.57	*0.92*	*to*	*7.15*		1.91	*0.69*	*to*	*5.31*	0.217
	*30–39 years old*	111	93	2.75	*1.12*	*to*	*6.75*		3.48	*1.35*	*to*	*9.01*	**0.010**
	*20–29 years old*	173	162	3.07	*1.23*	*to*	*7.67*		3.18	*1.25*	*to*	*8.07*	**0.015**
	*Under 20*	27	27	1.66	*0.62*	*to*	*4.46*		2.87	*0.99*	*to*	*8.33*	0.052
Mother/caregiver’s marital status								0.433					
	*Single*	29	36	1									
	*Divorced/widowed*	54	61	1.10	*0.60*	*to*	*2.02*						
	*Married*	264	248	1.32	*0.79*	*to*	*2.22*						
Mother/caregiver’s educational level								0.0157					
	*Primary*	130	164	1					1				
	*Secondary/tertiary*	67	65	1.30	*0.86*	*to*	*1.96*		1.21	*0.77*	*to*	*1.90*	0.412
	*Uneducated*	150	116	1.63	*1.17*	*to*	*2.28*		1.90	*1.33*	*to*	*2.71*	**< 0.001**
Mother/caregiver’s profession								**0.0075**					
	*Unemployed*	47	75	1					1				
	*Farmer/Breeder*	232	221	1.68	*1.11*	*to*	*2.52*		2.13	*1.37*	*to*	*3.31*	**0.001**
	*Employee*	68	49	2.22	*1.32*	*to*	*3.72*		2.34	*1.34*	*to*	*4.07*	**0.003**
Mother/caregiver’s religion								**0.0075**					
	*None/traditional*	16	8	1									
	*Christian*	306	327	0.47	*0.20*	*to*	*1.11*						
	*Muslim*	25	10	1.25	*0.41*	*to*	*3.84*						
Place of residence								**0.0141**					
	*Urban*	27	12	1					1				
	*Rural*	320	333	2.34	*1.17*	*to*	*4.70*		2.30	*1.09*	*to*	*4.84*	**0.029**
Sex of child								0.057					
	*Male*	165	189	1					1				
	*Female*	182	156	1.34	*0.99*	*to*	*1.80*		1.35	*0.99*	*to*	*1.85*	0.059
Place of birth of the child								**0.0024**					
	*Health facility*	46	22	1					1				
	*At home/on the road*	301	323	2.24	*1.32*	*to*	*3.82*		2.35	*1.35*	*to*	*4.09*	**0.002**

### Reasons for not vaccinating refugee and internally displaced children in DRC

#### Reasons related to people’s perceptions and feelings

The reasons related to people’s thinking and feelings, which may contribute to reducing parents’ motivation to have their children vaccinated (fear of side effects), were cited for half of ZD children (50.0%), higher than for over a third of those who were UV (38.3%) (Table 5).

**Table 5:** Reasons for non-vaccination of refugee and IDPs in the DRC in 2022 and comparison between zero-dose and under-vaccinated children

Variables		Proportion (% and 95% CI) among ZD children (*n* = 64)	Proportion (% and 95% CI) among UV children (*n* = 347)	*p*
Reasons related to people’s perceptions and feelings “Confidence in vaccine benefits”		50.0 (27.2–72.8)	38.3 (26.1–51.8)	NS
	Fear of side effects			
Reasons related to social processes “Family norms”		40.6 (28.5–53.6)	35.7 (30.7–41.0)	NS
	Family problems, relegating the importance of child vaccination			
Practical issues		90.5 (77.4–97.3)	97.1 (93.4–99.1)	NS
Factors limiting geographic accessibility		5.0 (0.1–24.9)	25.0 (14.7–37.9)	NS
	Vaccination site too far			
Intrinsic quality factors of health workers		20.0 (5.8–43.7)	16. 7 (8.3–28.5)	NS
	Poor reception of families by health workers			
Organizational factors of the health facility		88.1 (74.4–96.0)	93.7 (89.0-96.8)	NS
	Vaccination site not known by families	54.7 (41.8–67.2)	45.5 (40.2–50.9)	
	Long wait	20.0 (5.7–43.7)	36. 7 (24.6–50.1)	
	Vaccination schedule not respected	20.0 (5.7–43.7)	30.0 (18.9–43.2)	
	Frequently out of stock	10.1 (1.2–31.7)	5.0 (1.0-13.9)	

With: PSC = preschool consultation; n = number of subjects; 95% IC = 95% confidence interval, NS = non-significant.

### Reasons related to social processes

Reasons related to social processes (family problems, relegating the importance of child vaccination) were cited for 2 in 5 of ZD children (40.6%), higher than for UV children (35.7%) (Table 5).

### Programmatic and practical reasons

Almost all respondent cited having “practical issues” (reasons likely to limit geo-graphic access, those related to the intrinsic quality of health workers and organization of health facilities), with these reasons being cited by 90.5% of ZD children and by 97.1% of those who were UV (Table 5).

As for the results by sub-categories, the most cited reasons were those relating to the organization of health facilities (in 88.1%of ZD children, and 93.7% among the UV children. Next in line were reasons related to the interpersonal relational factors of health workers (poor reception of families by health workers) in 20.0% of zero-dose and 16.7% of UV children. Finally, “vaccination site too far” in 5% of ZD children and in 25% of UV children (25.0%) (Table 5).

## Discussion

The results of this survey showed that the percentage of ZD children aged 12 to 23 months was 9.3%, and that of under-vaccinated (UV) was 40.9%. The drop-out rate, calculated as Penta1-Penta3/Penta1 was of 34.9%. The proportion of ZD children among refugees and internally displaced persons is lower compared to that of the general population which is 19.1% according to the results of the 2021 vaccination coverage survey [10]. On the other hand, the proportion of UV children among refugees and internally displaced persons is much higher than that of the general population which is 25.5% according to the results of the same survey [14]. This situation suggests that more needs to be done to ensure completion of vaccination schedules, as these special populations seem to accept vaccination illustrated by a large majority starting the vaccination schedule without completing it. This is important as all basic doses are not only needed for pentavalent, but also for oral polio, pneumococcal conjugate vaccines and rotavirus. Furthermore, the high drop-out seen here also suggests that these children likely have not received their injectable polio vaccine (IPV) and any measles or yellow fever vaccine dose.

ZD children and UV in these special populations had a significant history of being born at home or on the road. For UV children, our study found that this status was significantly associated with mothers/caregivers aged under 40, who didn’t go to school and who had an occupation, residents in rural environment, in addition to being born outside a health facility. Place of birth as an explanatory factor had unfortunately not been researched in the general population [10]. However, several studies have high-lighted a link between the place of delivery and the vaccination status of the child, with women giving birth at home less likely to have their children vaccinated com-pared to those who giving birth in a health facility [15–17]. This could be explained by the fact that the latter would be exposed during pregnancy to advice on the im-portance of vaccinating children in a timely manner [18]. As for other factors, the low age of mothers/caregivers has also been found in the general population of the DRC according to the results of the 2021 vaccination coverage survey [10], and also in several studies conducted in other countries. The older the mother or childminder, the less hesitant she may be and the fewer obstacles she may encounter in having her child vaccinated [19–22]. In terms of educational level in the general population, on the other hand, mothers/caregivers of uneducated children and those with only primary or secondary education were more likely to have their child vaccinated than those with higher or university education [10]. Several researchers have also found a kind of inverse correlation between the mother’s level of education and the child’s non-vaccination. The higher the level of education, the less reluctant the mother is to have her child vaccinated [15, 23–30]. This is not the case in our study and would probably be based on the behavioral changes that education would bring, such as changes in attitudes, traditions and beliefs, or greater autonomy and control over household resources that would improve health care seeking [15, 23–30].

The professional occupation of the mother/caregiver (farmer, herder, employee) was not identified as an associated factor in the general population of the DRC [10]. On the other hand, cross-sectional studies in other countries, notably Ethiopia, have shown a significant link between the mother’s gainful occupation and the child’s immunization status. However, the proportion of unvaccinated children was significantly higher among mothers who were only housewives [26, 31]. This is not the case in our study, and is probably due to the lack of time associated with these occupations. And in terms of place of residence, our results corroborate those of several other researchers. Studies in Kenya and Nigeria, for example, have also found that children living in rural areas are less likely to be vaccinated than those living in urban areas [15, 29]. This may be explained by the fact that knowledge of the benefits of vaccination is lower in rural than in urban areas [15, 29].

As regards the reasons for non-vaccination of ZD and under-vaccinated children in this special population, reasons related to people’s thoughts and feelings, as well as social processes that can reduce parents’ motivation to have their children vaccinated, were cited more for ZD children than for under-vaccinated. These reasons included fear of side effects, family problems, relegating the importance of child vaccination. On the other hand, programmatic and practical reasons were cited more by under-vaccinated children than by ZDs. These reasons included vaccination site too far, poor reception of families by health workers, vaccination site not known by families, long wait and vaccination schedule not respected, frequently out of stock. These trends are virtually the same as those found in the general population [14]. For the reasons behind the very high proportion of under-vaccinated children, previous negative experiences when vaccinating a child can shed light on the perceived problematic nature of practical obstacles. They can affect even families who were motivated to have their children vaccinated. These children would probably have been fully vaccinated if health facilities had facilitated, rather than blocked, their access to vaccines. Several other studies carried out in developing countries have also found the same reasons linked to the organization of health structures to be at the root of children’s non-vaccination [32–48].

## Limitations

This study has several limitations. It reflects cross-sectional data collection at a specific moment and does not provide longitudinal data collection with a high risk of selection bias and residual confounding. The sample size was small, limiting the power to identify differences that may be important but that in our study would not reach statistical significance. Other limitations relate to the consideration of recall responses in the estimation of different coverages, which may underlie memory or social desirability bias. In addition, refugee and IDP sites not included in the sampling frame may have been excluded, and these sites may also have their own reasons for non-vaccination. There is a possibility of misclassification of results of interest, as the survey relied on maps and recall when a map was not available, and map availability varied considerably between sites.

## Conclusions

A sizeable proportion of children are ZD and UV in refugee and IDP sites in the DRC. This proportion is particularly high for UV children, with a very high drop-out rate resulting on immunity gaps. Children not born in a health facility were more likely to be ZD while caregivers aged 20 to 39, lack of maternal education, being a farmer, employee, living in rural area and not born in a health facility were mostly linked to a child being UV. This study also revealed that the reasons for ZD children were more often linked to challenges in motivating parents or caregivers to vaccinate, while for UV children, the reasons for not completing their vaccination schedule were more often linked to programmatic and practical problems in the health system. In this way, these results can be used to help decision-makers better prepare and implement effective interventions to improve vaccine coverage, and completion of vaccination schedules among refugees and IDPs in the DRC and achieve the goal of leaving no one behind.

## Data Availability

No datasets were generated or analysed during the current study.

## Abbreviations

DRC: Democratic Republic of the Congo
CI: Confidence interval
ZD: Zero-dose
UV: Under-vaccinated
IDPs: Internally displaced persons
SDGs: Sustainable Development Goals
VCS: Vaccination coverage survey
CNES: National Health Ethics Committee
n: Number of subjects
SD: Standard Deviation
WHO: World Health Organization
p: p-value
